# Progress in cytokine research for ARDS: A comprehensive review

**DOI:** 10.1515/med-2024-1076

**Published:** 2024-10-28

**Authors:** Kaihuan Zhou, Junyu Lu

**Affiliations:** Intensive Care Unit, The Second Affiliated Hospital of Guangxi Medical University, Nanning, Guangxi 530007, China; Intensive Care Unit, The Second Affiliated Hospital of Guangxi Medical University, No. 166 Daxuedong Road, Nanning, Guangxi 530007, China

**Keywords:** ARDS, IL-6, pathogenesis, cytokines, immunity, targeted therapy

## Abstract

**Introduction:**

Acute respiratory distress syndrome (ARDS) is a critical form of acute respiratory failure characterized by diffuse alveolar damage, refractory hypoxemia, and non-cardiogenic pulmonary edema, resulting in high mortality. Dysregulated inflammation, driven by cytokines, is central to ARDS pathogenesis, progression, and prognosis.

**Objective:**

This review synthesizes current knowledge on the role of cytokines in ARDS and evaluates their potential as therapeutic targets, offering new insights for clinical management.

**Methods:**

A comprehensive analysis of recent studies was conducted to explore the roles of pro-inflammatory cytokines (e.g., IL-1β, IL-6, IL-8) and anti-inflammatory cytokines (e.g., IL-10, IL-22) in ARDS pathogenesis and to assess current and emerging therapies targeting these cytokines.

**Results:**

Pro-inflammatory cytokines are crucial in initiating inflammatory responses and lung injury in early ARDS, while anti-inflammatory cytokines help regulate and resolve inflammation. Targeted therapies, such as IL-1 and IL-6 inhibitors, show potential in managing ARDS, particularly in COVID-19, but their clinical efficacy is still debated. Combination therapy strategies may enhance outcomes, but further large-scale, multicenter randomized controlled trials are required to establish their safety and efficacy.

**Conclusion:**

Understanding cytokine regulation in ARDS could lead to innovative therapeutic approaches. Future research should focus on cytokine roles across ARDS subtypes and stages and develop biomarker-driven, individualized treatments.

## 1 Introduction

Acute respiratory distress syndrome (ARDS) is characterized by refractory hypoxemia and non-cardiogenic pulmonary edema, resulting from diffuse alveolar damage. The primary pathophysiological feature of ARDS is increased permeability of the alveolar-capillary barrier due to widespread damage to the alveolar epithelium and vascular endothelium [1]. This damage leads to pulmonary interstitial edema, increased respiratory workload, impaired gas exchange, and, ultimately, acute respiratory failure. The ARDS mortality rate remains high, with global rates ranging from 30 to 50% [2,3]. The recent coronavirus disease (COVID-19) pandemic has significantly increased the prevalence of ARDS, highlighting the severity of this condition and the urgent need for further research. Although supportive treatment strategies, such as lung-protective ventilation and extracorporeal membrane oxygenation, have improved patient outcomes to some extent, no effective pharmacological treatments are available for this high-mortality disease.

Cardiogenic pulmonary edema is primarily caused by heart failure and is characterized by hemodynamic abnormalities and fluid retention. In contrast, non-cardiogenic pulmonary edema, such as ARDS, results from inflammation-mediated damage to the pulmonary barrier. The main issue in cardiogenic pulmonary edema is hemodynamic imbalance – where certain cytokines (e.g., IL-6 and TGF-β) play relatively minor roles [4]. However, non-cardiogenic pulmonary edema, including ARDS, is driven by inflammation-induced lung barrier damage, with pro-inflammatory cytokines (e.g., tumor necrosis factor-α [TNF-α], IL-1β, and IL-6) playing critical roles in propagating inflammation and exacerbating lung injury [5,6].

In the pathogenesis of ARDS, cytokines are key contributors to inflammatory cascades and the resulting epithelial and endothelial injury, making them important drivers of disease progression and potential therapeutic targets [1]. This review explores recent advances in the study of cytokines related to ARDS, focusing on their roles in disease progression and their therapeutic potential, with the aim of providing new insights for clinical treatment.

## 2 Immune dysregulation: A pivotal indicator of ARDS

ARDS is a rapidly progressing condition characterized by diffuse bilateral lung injury and refractory hypoxemia caused by non-cardiogenic pulmonary edema. According to the Berlin definition [7] and the 2023 global ARDS definition [8], common causes of ARDS include pulmonary infections, sepsis, severe trauma, and aspiration. The primary mechanisms of ARDS involve inflammatory imbalance, increased permeability of the pulmonary endothelium and epithelium, and diffuse alveolar damage. An imbalance between pro-inflammatory and anti-inflammatory factors is a hallmark of ARDS pathophysiology [1,9].

### 2.1 Activation of effector cells

Both pulmonary and extrapulmonary factors can cause direct and indirect lung injury, leading to the damage of vascular endothelial and alveolar epithelial cells through various mechanisms. This damage activates effector cells, such as macrophages, neutrophils, and effector T cells. The activation of these cells has distinct clinical and pathophysiological implications for the lungs and alveoli. In the early stages of ARDS, neutrophils, as central drivers of the inflammatory response, play a critical role in damaging the alveolar epithelium and vascular endothelium [10].

Neutrophils, as the first line of defense in the innate immune response, are essential for pathogen defense through their rapid and robust inflammatory response. However, when neutrophils become excessively accumulated and persistently activated, their destructive effects on alveolar and vascular structures far outweigh their protective functions [11]. Early in ARDS, neutrophils rapidly accumulate in the alveoli and lung interstitium under the guidance of chemokines, releasing large amounts of reactive oxygen species (ROS), reactive nitrogen species (RNS), proteases (such as elastase), and pro-inflammatory cytokines (such as IL-6). These mediators directly compromise the integrity of alveolar epithelial and vascular endothelial cells, severely impairing the barrier function of the alveoli and vasculature and increasing vascular permeability [1,12].

The excessive production of ROS and RNS is a hallmark of the pathophysiological process of ARDS. These reactive molecules cause oxidative damage to cell membranes, proteins, and DNA, disrupting cellular function and integrity and further impairing the barrier function of the alveoli and vasculature [13,14,15]. Oxidative stress not only directly damages lung structures but also promotes the release of additional pro-inflammatory cytokines by activating signaling pathways, such as nuclear factor-kappa B and mitogen-activated protein kinases, amplifying the local inflammatory response [16,17]. Moreover, chemokines released by neutrophils recruit and activate effector T cells, exacerbating inflammation and leading to extensive non-specific tissue damage [12,18].

**Figure 1:**
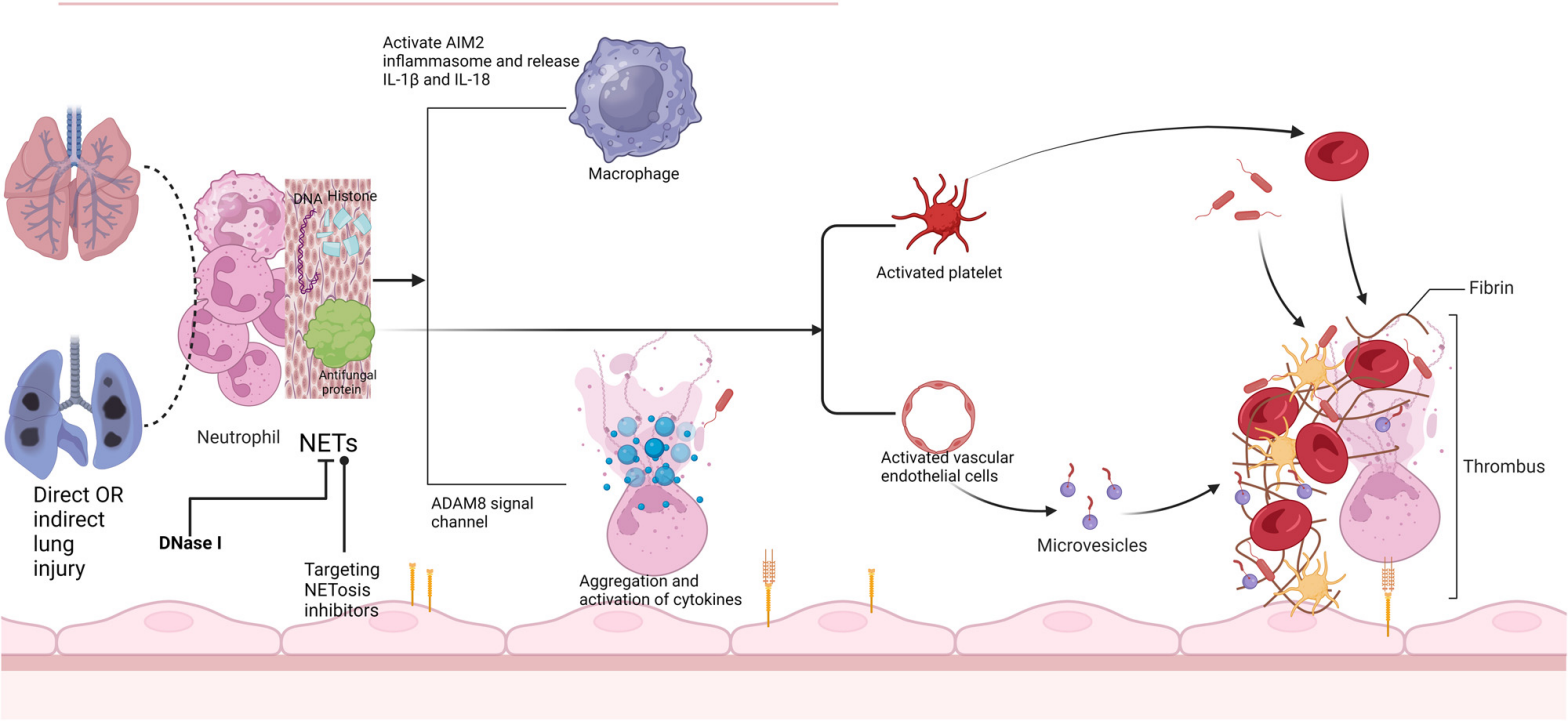
Key mechanisms by which neutrophils and NETs contribute to the pathogenesis of ARDS. During lung injury, neutrophils become activated and release NETs, which are composed of DNA, histones, and antimicrobial proteins. While NETs capture pathogens as part of the immune response, they also cause tissue damage. NETs activate AIM2 inflammasomes, prompting macrophages to release pro-inflammatory cytokines, such as IL-1β and IL-18, which intensify the inflammatory response. Furthermore, NETs upregulate the expression of pro-inflammatory mediators via the ADAM8 pathway, leading to additional activation of inflammatory cells. NETs also interact with endothelial cells and platelets, promoting thrombosis. The figure also presents targeted therapeutic strategies, including the degradation of NETs using DNase I and the application of NETosis inhibitors, which may help mitigate inflammation and tissue damage, providing new potential approaches for the treatment of ARDS and COVID-19. NETs, neutrophil extracellular traps; COVID-19, coronavirus disease.

Recent studies have identified the formation of neutrophil extracellular traps (NETs) as an important factor in the pathogenesis of ARDS [19]. NETs, composed of DNA, histones, and antimicrobial proteins released by neutrophils, capture pathogens and participate in immune defense. However, excessive NET formation can trigger inflammatory responses and worsen tissue injury [20]. NETs promote lung damage by activating AIM2 inflammasomes and ADAM8 signaling pathways, driving macrophage activation, and enhancing the expression of pro-inflammatory cytokines [21,22]. In COVID-19-related ARDS, NET formation is particularly prominent; it not only captures the virus but also activates endothelial cells and platelets, promoting thrombosis (Figure 1) [23]. Additionally, neutrophils in COVID-19-related ARDS exhibit specific activation states, such as interferon-activated neutrophils (IFNactive) and prostaglandin-activated neutrophils (PGactive), which further intensify inflammation and tissue injury through interferon and prostaglandin signaling pathways [24]. NET formation is closely associated with disease severity in patients with COVID-19 [25]. Post-pandemic research targeting NETs may offer new directions for reducing mortality in COVID-19-related ARDS, such as using DNase I to degrade NETs or employing NETosis inhibitors to mitigate excessive inflammation caused by NETs [26].

### 2.2 Immune imbalance

Early acute lung injury (ALI) in ARDS is driven by dysregulated inflammatory responses, characterized by both local and systemic acute inflammation. While a moderate inflammatory response can provide protective effects, an imbalance between pro-inflammatory and anti-inflammatory systems may lead to severe tissue damage. Pathogen-associated molecular patterns (PAMPs) or damage-associated molecular patterns (DAMPs) bind to toll-like receptors on alveolar epithelial cells and macrophages, thereby activating the innate immune system. This activation shifts macrophages toward a pro-inflammatory phenotype, resulting in the release of pro-inflammatory cytokines and neutrophil chemokines, such as IL-8, which promote the progression of ARDS [1].

Excessive release of inflammatory mediators can trigger a cytokine storm. Cytokines are crucial for coordinating antimicrobial effector cells and regulating signals to guide, amplify, and resolve the immune response. However, excessive cytokine production may lead to uncontrolled inflammation and multi-organ failure. Elevated levels of inflammatory mediators, such as IL-6, IL-8, and TNF, induce chemotaxis and activation of neutrophils and macrophages, which then release various damaging mediators [27]. These mediators include ROS, proteases, and pro-inflammatory derivatives like prostaglandins and leukotrienes, causing damage to capillary endothelial cells and alveolar epithelial cells. Ultimately, this cascade impairs gas exchange, leading to respiratory failure and refractory hypoxemia in patients with ARDS.

## 3 ARDS-related cytokines

### 3.1 Pro-inflammatory cytokines

#### 3.1.1 IL-1β

IL-1β plays a pivotal role in the development and progression of ARDS. Primarily secreted by macrophages, IL-1β functions in the inflammatory environment through both paracrine and autocrine mechanisms. It activates the IL-1 receptor (IL-1R) expressed on various cell types and stimulates the production of several chemokines, such as IL-8 and monocyte chemoattractant proteins, contributing to increased vascular endothelial permeability and fluid accumulation [28,29]. Research has shown that IL-1β exacerbates sepsis-induced ALI by inhibiting VE-cadherin transcription [30]. In a biomarker-based mortality risk model for ARDS, IL-1β levels were negatively correlated with patient prognosis [31]. Wu et al. demonstrated that caspase-8 activation could inhibit IL-1β through Fadd +/−Ripk3−/−BMDCs, identifying a potential new therapeutic target for ARDS [32].

#### 3.1.2 IL-6

IL-6 is a multifunctional cytokine produced by various cell types, playing a significant role in infection, immunity, tumor development, and inflammation [33,34]. In ARDS, IL-6 is primarily secreted by mononuclear macrophages and endothelial cells, which become activated during the inflammatory response. IL-6 amplifies both local and systemic inflammation, thereby exacerbating the pathological process of ARDS [35]. Research has shown that IL-6 exerts its biological effects by forming a complex with the IL-6 receptor, which then binds to the ubiquitously expressed glycoprotein 130 (gp130) [36]. Additionally, IL-6 promotes lung inflammation and injury by activating the JAK2/STAT3 signaling pathway [37]. Several studies have demonstrated that IL-6 is a key initiator of the cytokine storm in COVID-19-related lung injury, with IL-6 levels directly correlated with patient outcomes. Thus, IL-6 serves as a critical biomarker for predicting disease severity and prognosis in patients with ARDS [38,39,40]. Given its pivotal role, the IL-6 inhibitor tocilizumab has been widely used in treating COVID-19-related ARDS [41].

#### 3.1.3 IL-8

IL-8, a potent neutrophil chemotactic factor expressed by various cell types, including neutrophils, fibroblasts, epithelial cells, hepatocytes, alveolar macrophages, and endothelial cells, plays a significant role under multiple pathophysiological conditions [42]. In ARDS, IL-8 is primarily secreted by macrophages and lung epithelial cells that become activated upon recognizing DAMPs or PAMPs, shifting to a pro-inflammatory phenotype [1]. Studies have shown that high concentrations of IL-8 are present in the bronchoalveolar lavage fluid (BALF) and pulmonary edema fluid of patients with ALI or ARDS, with BALF IL-8 levels significantly associated with mortality in sepsis, ALI, and ARDS [31,43]. This pro-inflammatory effect may result from dysregulation of the IL-8/CXCR1/2 signaling axis [44]. Additionally, elevated plasma IL-8 levels are indicative of poor prognosis in patients with ARDS [31]. In COVID-19-related ARDS, IL-8 acts as a key chemokine that induces neutrophil activation and aggregation, thereby exacerbating lung injury [44]. Further investigation into the pathogenic mechanisms of IL-8 in the post-pandemic era may reveal new therapeutic targets for ARDS [45].

#### 3.1.4 IL-33

IL-33 is a nuclear cytokine of the IL-1 family that acts as an “alarm signal” during various infections, exerting its biological function through the IL-33/ST2 axis [46]. In ARDS, IL-33 is released extracellularly when lung epithelial cells are damaged, playing a pro-inflammatory role [47]. Basic research has shown that IL-33 promotes lung inflammation by activating STAT3 in alveolar macrophages, enhancing the expression of matrix metalloproteinases MMP2 and MMP9 [48]. In a mouse model of lung inflammation/injury, Fu et al. found that IL-33 levels in serum, BALF, and lung tissue were significantly higher 24 h post-administration than in the control group, suggesting that IL-33 may be a key factor in ARDS development [49]. Other research indicates that reducing IL-33 concentrations in mouse models of ALI/ARDS can control lung inflammation by inducing a Th17 response, affecting the Th17/Treg balance, and regulating autophagy, thereby improving prognosis [50,51]. Current evidence suggests that IL-33 plays an important role in ALI and may become a new diagnostic marker for ARDS. Further investigation into IL-33 may uncover novel treatment options for ARDS [48].

#### 3.1.5 High mobility group box 1 (HMGB1)

The HMGB1 is a highly conserved protein that functions as both a nuclear and membrane-associated protein. Membrane-bound HMGB1 plays a crucial role in the late stages of inflammation by promoting the recruitment and chemotaxis of inflammatory cytokines, including TNF, IL-1, and IL-6 [52]. During ARDS progression, neutrophils and macrophages are major sources of HMGB1. The release of HMGB1 helps initiate and amplify local inflammatory responses, leading to pulmonary edema and worsening the clinical course of ARDS [53]. In a lipopolysaccharide (LPS)-induced ALI mouse model, significant increases in plasma and tissue HMGB1 levels have been reported [54]. Li et al. demonstrated that the HMGB1/PI3K/Akt/mTOR signaling pathway regulates dendritic cell maturation and function, contributing to the pathogenesis of ALI in mouse models [55]. Furthermore, studies have shown that blocking the HMGB1 signaling pathway can alleviate ALI and improve prognosis in mice [56]. During the COVID-19 pandemic, HMGB1 was identified as a potential therapeutic target for COVID-19-related lung injury and could also serve as a key cytokine for predicting ARDS severity [57].

### 3.2 Anti-inflammatory cytokines

#### 3.2.1 IL-1 receptor antagonist (IL-1 RA)

In ARDS, while multiple inflammatory factors are released, several endogenous anti-inflammatory mediators, such as IL-10 and IL-1 RA, are also produced. IL-1RA is a naturally occurring inhibitor of IL-1β that lacks agonist activity but competitively binds to IL-1 receptors, playing a crucial role in various diseases, including rheumatoid arthritis and inflammatory bowel disease [58,59]. During inflammatory responses, activated monocytes and macrophages secrete IL-1RA to regulate and limit IL-1-triggered inflammation, significantly reducing overall inflammatory levels in the body [60,61]. However, Dahmer et al. found that high levels of IL-1RA were positively correlated with poor outcomes in pediatric ARDS. Additionally, high IL-1RA levels were significantly associated with disease progression, oxygenation, and long-term prognosis, potentially due to an imbalance between pro-inflammatory and anti-inflammatory factors in the lungs [62].

#### 3.2.2 IL-10

IL-10 is a key anti-inflammatory mediator, primarily derived from macrophages and regulatory T cells. It plays a vital role in protecting the host from excessive responses to pathogens and the microbiome while also contributing to wound healing and autoimmune regulation [63]. In ARDS, IL-10 is mainly secreted by macrophages and can inhibit the production of pro-inflammatory cytokines, thereby reducing lung inflammation [64]. Additionally, IL-10 can modulate the Fth1^hi^ Neu subtype, enhancing its antioxidant capacity and reducing apoptosis [11]. IL-10 also helps maintain inflammatory homeostasis by suppressing excessive inflammatory responses and upregulating immune function [63]. A recent study showed that increased IL-10 expression can mitigate lung injury caused by sepsis [65]. Shih et al. suggested that IL-10 is a critical anti-viral and anti-fibrotic cytokine, which can alleviate the severity of ALI/ARDS [64].

#### 3.2.3 IL-22

IL-22 is a member of the IL-10 subfamily, primarily produced by T cells and innate lymphoid cells, with roles in anti-infection, anti-tumor activity, and tissue repair [63]. IL-22 is particularly involved in the later stages of ARDS, where it contributes to tissue repair processes. During immune responses in the lung, innate lymphoid cells secrete IL-22 to maintain epithelial barrier integrity and promote tissue recovery and regeneration following lung infection or injury [66]. IL-22 does not act directly on immune cells; rather, it regulates immunity through downstream signaling pathways. It protects the intestinal epithelium, enhances mucosal barrier function, and directly strengthens the defense capabilities of tissue cells to prevent pathogen invasion [63,67,68]. Additionally, studies have confirmed that IL-22 can reduce inflammation and vascular leakage in ALI, thereby slowing the progression of ARDS [69].

#### 3.2.4 IL-13

IL-13 is an important anti-inflammatory cytokine, mainly derived from type 2 innate lymphoid cells residing in tissues. It plays a crucial role in autoimmune diseases, cellular senescence, and wound-healing processes [70]. In ARDS, IL-13 is primarily secreted by ST2+ regulatory T cells (Tregs) stimulated by IL-33. It regulates local inflammatory responses and reduces ALI by inducing the gene expression of reparative or immunomodulatory myeloid cells [71]. Bhandari et al. found that mice lacking IL-13 expression were more susceptible to developing ALI and had a higher mortality rate than normal mice, suggesting a protective role of IL-13 in the course of ARDS [72]. Moreover, Liu et al. demonstrated that IL-13 secretion can inhibit lung inflammation, exert anti-inflammatory effects, and control disease progression in ALI mice [71]. Other reports indicate that IL-13 promotes alveolar epithelial cell regeneration by stimulating macrophage proliferation [73]. Recent studies suggest that IL-13 enhances neutrophil apoptosis and reduces inflammatory damage through the STAT3 and STAT6 signaling pathways, thereby improving lung injury [74]. A wealth of evidence indicates that IL-13 is closely associated with the prognosis of ALI/ARDS.

### 3.3 Chemokines

Chemokines are a specialized class of cytokines renowned for their ability to regulate immune cell migration [75]. In the pathophysiology of ARDS, chemokines promote local inflammatory responses by directing immune cells to the injured areas of the lung [1]. For example, monocyte chemoattractant protein-1 (MCP-1) recruits monocytes and neutrophils through the CCL2–CCR2 chemokine axis, and their excessive accumulation and activation further exacerbate lung injury [76,77].

However, the role of chemokines extends beyond cell migration; they are also involved in more complex processes of immune regulation and tissue repair [75]. By activating multiple signaling pathways, chemokines influence the function and polarization of inflammatory cells and participate in tissue regeneration and fibrosis regulation at different stages of the disease. For instance, CXCL12 can regulate epithelial cell survival and reduce apoptosis, providing protection to lung tissue [78]. Nonetheless, persistent inflammatory stimuli may cause abnormally high expression of chemokines, which, in turn, promotes fibroblast proliferation and the fibrotic process, ultimately affecting lung function recovery in patients with ARDS [79].

Additionally, chemokines influence immune balance in ARDS by modulating T-cell polarization and function. For example, CCL5 and CXCL10 play key roles in regulating Th1/Th2 balance [80], and different combinations of chemokines can modulate the type of immune response, thereby affecting the intensity and duration of inflammation. Chemokines are also crucial in the polarization of macrophages into M1 or M2 phenotypes [81]. M1 macrophages, influenced by certain chemokines, primarily mediate inflammatory responses and exacerbate tissue damage by releasing pro-inflammatory mediators. In contrast, M2 macrophages are involved in anti-inflammatory and tissue repair processes, promoting tissue regeneration and fibrosis during the later stages of inflammation [82].

Given these mechanisms, chemokines and their receptors are being considered potential therapeutic targets for ARDS [83]. Recent research has explored the use of nanomedicine to target lung macrophages and regulate chemokine expression as a treatment strategy for ARDS [84,85,86]. A deeper understanding of the multifaceted roles of chemokines may lead to the development of more effective therapeutic strategies for ARDS in the future.

## 4 Cytokine-related therapies for ARDS

### 4.1 IL-1 inhibitors

IL-1 is a prototypical pro-inflammatory cytokine composed primarily of two ligands: IL-1α and IL-1β, with IL-1β playing the key role in promoting inflammation. As mentioned earlier, IL-1β is the primary driver of the pro-inflammatory response. Anakinra and canakinumab are IL-1β inhibitors currently used clinically to treat autoimmune diseases such as rheumatoid arthritis and early-onset multisystem inflammatory diseases [87,88]. Recent studies have shown that anakinra can reduce mortality in patients with cytokine storms caused by sepsis [89]. Additionally, several studies have demonstrated that anakinra can improve respiratory function, reduce the need for mechanical ventilation, and lower mortality rates in patients with COVID-19-related ARDS [90,91]. Although these results are promising, the studies involved relatively small sample sizes. Larger randomized controlled trials (RCTs) are needed to further confirm the reliability, safety, and efficacy of these treatments.

### 4.2 IL-6 inhibitors

IL-6 is a critical inflammatory factor in the development of ARDS. Several IL-6 inhibitors, such as tocilizumab and sarilumab, have been used to treat autoimmune diseases and to suppress cytokine storms, demonstrating efficacy in conditions like rheumatoid arthritis, systemic sclerosis, and sinusitis [92,93]. Among these, tocilizumab is the most thoroughly studied IL-6 inhibitor for ARDS. Tocilizumab is a monoclonal antibody that specifically blocks the IL-6 receptor, thereby reducing the inflammatory response. Terzi et al. showed that tocilizumab can downregulate the expression of pro-inflammatory cytokines, including TNF-α, IL-1β, IL-6, and IL-8, effectively preventing cytokine storms and exerting antioxidant effects [94]. Case reports have documented the successful use of tocilizumab in treating patients with COVID-19-related ARDS [95,96]. Additionally, retrospective studies have indicated that tocilizumab can significantly reduce mechanical ventilation duration and mortality in patients with ARDS, improving patient outcomes [97]. Furthermore, RCTs have demonstrated that tocilizumab can reduce systemic inflammatory responses and improve clinical outcomes in patients with COVID-19-related ARDS [98,99,100]. Meta-analyses have further confirmed that, compared to standard treatments and placebos, IL-6 inhibitors can lower the 28-day all-cause mortality in patients with COVID-19-related ARDS [101].

### 4.3 Other cytokine-related therapies

In addition to IL-1 and IL-6 receptor inhibitors, other drugs targeting cytokine-mediated inflammatory pathways have gradually entered clinical use. These include emapalumab, an interferon-γ inhibitor, and Janus kinase (JAK) inhibitors like ruxolitinib and baricitinib, which block the JAK/STAT signaling pathway [102]. However, the efficacy and safety of these drugs for treating ARDS remain unproven, as they lack validation in RCTs. As more research evidence emerges, new cytokine inhibitors, including tocilizumab, anakinra, and baricitinib, show promise as potential options for treating severe ARDS.

## 5 Conclusion and future perspectives

**Figure 2:**
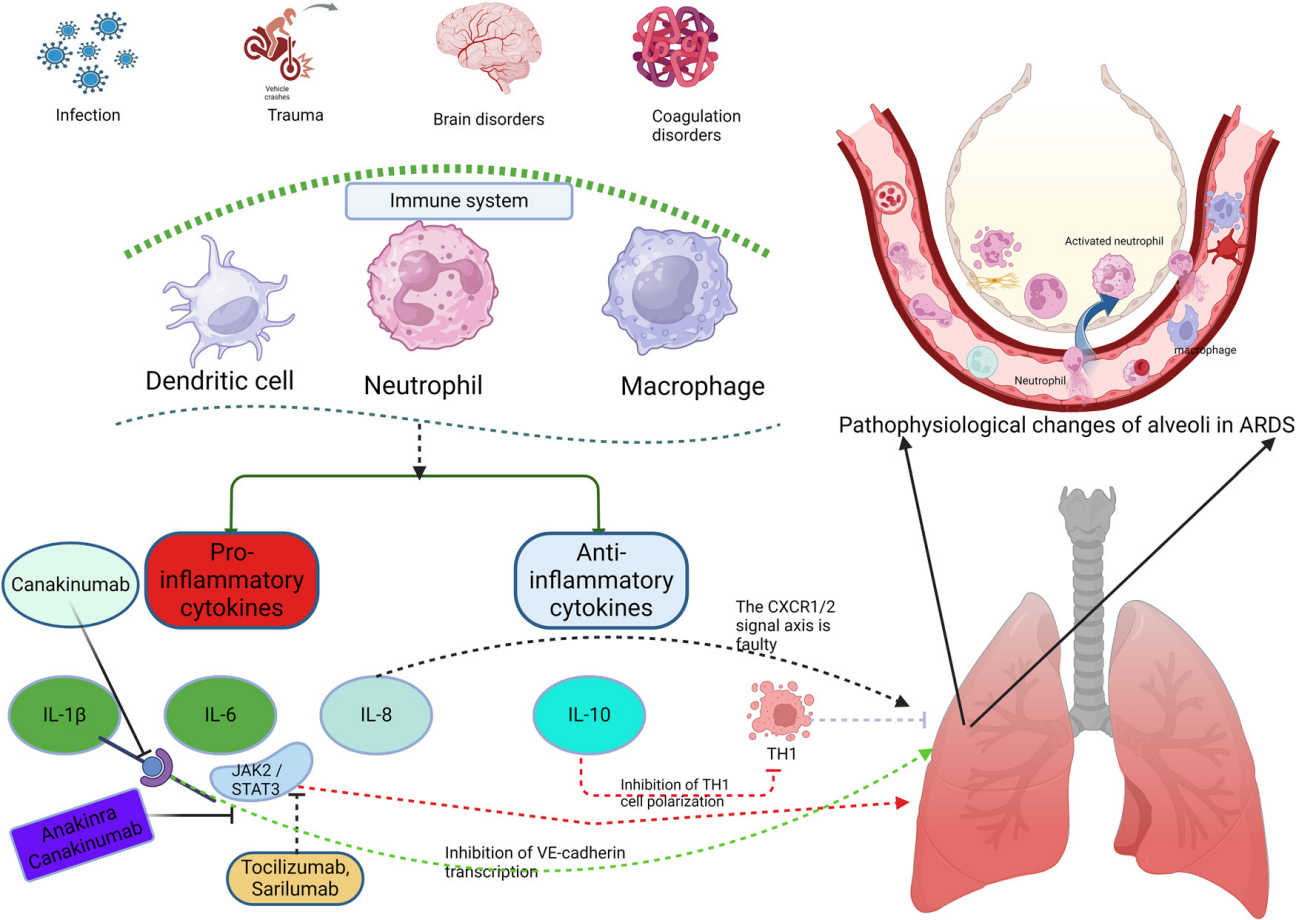
Key cytokines involved in the pathophysiology of ARDS and their therapeutic agents. Various conditions, such as infections and trauma, activate the immune system, leading to the release of both pro-inflammatory cytokines (e.g., IL-1β, IL-6, and IL-8) and anti-inflammatory cytokines (e.g., IL-10) by the monocyte-macrophage system. IL-1β contributes to ARDS development by inhibiting VE-cadherin transcription; IL-6 promotes ARDS through the JAK2/STAT3 pathway; and IL-8 facilitates ARDS progression via the CXCR1/2 signaling axis. Conversely, IL-10 exerts an anti-inflammatory effect by suppressing Th1 cell transcription. Several therapeutic agents targeting these cytokines have gradually been applied in clinical practice, including IL-1β inhibitors, such as canakinumab and anakinra, and the IL-6 inhibitor tocilizumab.

ARDS is a severe clinical syndrome characterized by high mortality and a lack of specific therapeutic options, driven by multiple factors. Its pathogenesis primarily involves immune dysregulation and an imbalance between pro-inflammatory and anti-inflammatory factors, with cytokines playing distinct roles during the acute and recovery phases of ARDS. This review summarizes the main cytokines involved in ARDS pathogenesis and their related therapeutic agents (Figure 2), highlighting early pro-inflammatory cytokines (such as IL-1β and IL-6), protective anti-inflammatory cytokines (such as IL-10 and IL-13), and the critical role of chemokines in regulating the inflammatory response and immune balance. As previously discussed, pro-inflammatory cytokines, such as IL-1β and IL-6, exacerbate lung injury during the acute phase of ARDS by activating inflammatory pathways. Conversely, anti-inflammatory cytokines, including IL-10, IL-13, and various chemokines, play protective roles at specific stages. These dual roles are crucial for disease progression and prognosis assessment.

Currently, cytokine-targeted therapies show considerable potential in treating ARDS. IL-6 inhibitors, such as tocilizumab, have demonstrated the ability to reduce systemic inflammatory responses and improve survival rates and other clinical outcomes in patients with severe COVID-19 in multiple RCTs [99,100]. However, the clinical benefits of other cytokine-targeted therapies remain controversial. For instance, IL-1β inhibitors have shown efficacy in reducing lung injury in animal models [104]; however, their clinical effectiveness varies widely among individuals. While observational and cohort studies suggest that these inhibitors may suppress cytokine storms and improve clinical outcomes in patients with COVID-19-related ARDS [90,91,105], RCTs and meta-analyses have generally yielded disappointing results [103,106]. Similarly, although early studies suggested that targeting the pro-inflammatory factor TNF could modulate immune responses [107] and that anti-TNF therapies might reduce cytokine release and improve respiratory function in animal models [108], clinical validation results have been less promising. Clinical studies have shown that anti-TNF drugs do not significantly improve outcomes in patients with COVID-19-related ARDS [109,110]. These findings indicate that, despite the theoretical advantages of anti-cytokine therapy, their clinical efficacy may vary significantly due to individual patient differences and the complexity of the disease, necessitating more precise applications based on specific patient characteristics (Table 1).

**Table 1:** New options for cytokine therapy

Cytokine	Study	Type of study	Secreting cells	Results	Drug/intervention	Main conclusion
IL-1β	Xiong et al. [30]	Animal model	Macrophages/endothelial cells	IL-1β impairs the pulmonary endothelial barrier by downregulating the cAMP-CREB-VE-cadherin pathway; the use of an IL-1R antagonist restores VE-cadherin levels to approximately 80% of normal (*p* < 0.05)	IL-1β Inhibitor	IL-1β inhibitors help maintain vascular endothelial integrity and reduce inflammatory damage
	Cavalli et al. [90]	Retrospective cohort study		In the Anakinra treatment group, compared to the control group, IL-1 levels, C REACTIVE PROTEIN levels, and ferritin levels decreased by approximately 60, 50, and 45%, respectively (*p* < 0.05)	Anakinra	Anakinra improves respiratory function and clinical outcomes in patients with COVID-19-related ARDS
	Declercq et al. [103]	RCT		Clinical improvement time for Anakinra vs no control group: 12 days (10–16) vs 12 days (10–15), HR 0.94, 95% CI 0.73–1.21. The clinical improvement rate at 28 days was 75% vs 73% (*p* = 0.63)		Anakinra does not significantly improve prognosis
IL-6	Terzi et al. [94]	Animal model	Macrophages and endothelial cells	In the tocilizumab group, levels of IL-6, IL-8, and IL-1β were significantly reduced by approximately 70, 55, and 60%, respectively, compared to the OA group (*p* < 0.05)	Tocilizumab	Tocilizumab downregulates the expression of pro-inflammatory cytokines TNF-α, IL-1β, IL-6, and IL-8, effectively preventing cytokine storms
	Hu et al. [97]	Retrospective cohort study		Compared to the group not treated with tocilizumab, the tocilizumab group had a lower mortality rate (2.00% vs 6.06%, HR 0.103, 95% CI, 0.013 to 0.798; *P* = 0.030) and showed a significant reduction in C REACTIVE PROTEIN and PROCALCITONIN levels		Tocilizumab significantly reduces IL-6 levels and gradually improves patients’ immune function within 14 days, leading to better clinical outcomes
	Soin et al. [98]	Randomized controlled trial		Mortality rate: tocilizumab group 31% vs standard care group 35% (RR 0.85, 95% CI 0.76-0.94, *p* = 0.0028)		Tocilizumab improves survival rates and other clinical outcomes
				Discharge rate: tocilizumab group 57% vs standard care group 50% (RR 1.22, *p* < 0.0001)		
				Invasive mechanical ventilation rate: tocilizumab group 35% vs standard care group 42% (HR 0.84, 95% CI 0.77–0.92, *p* < 0.0001)		
IL-8	Kaiser et al. [44]	Animal model	Macrophages and alveolar epithelial cells	Targeting the IL-8-CXCR1/2 axis reduces microthrombus formation in mice with severe ARDS	Blocking IL-8 signaling	Reveals targeting the IL-8 axis as a therapeutic target for ARDS
	Bime et al. [31]	Retrospective analysis		Patients with higher IL-8 levels had a 28-day mortality rate of 32%, significantly higher than the 19% observed in patients with lower IL-8 levels (*p* = 0.04)	—	Elevated IL-8 levels are associated with poorer clinical outcomes in ARDS patients
IL-33	Cheng et al. [51]	Animal model	Alveolar epithelial cells	Mice lacking IL-33 exhibit a lower proportion of inflammatory cells (such as Th17 cells), reduced levels of inflammatory cytokines (such as IL-6 and IL-17) in the BALF, and an improved Th17/Treg (regulatory T cell) balance	—	The deficiency of IL-33 leads to a reduced proportion of Th17 cells and lower levels of pro-inflammatory cytokines, thereby alleviating lung inflammation and demonstrating IL-33’s key role as a pro-inflammatory factor
HMGB1	Li et al. [55]	Animal model	Neutrophil and macrophages	HMGB1 significantly upregulates the expression of PI3K, Akt, and mTOR, promoting the maturation of dendritic cells (DCs) and the secretion of inflammatory cytokines; treatment with anti-HMGB1 or LY294002 significantly reduces the inflammatory response (*p* < 0.05)	Anti-HMGB1 antibodies or PI3K inhibitors	Following intervention, the activation of the PI3K/Akt/mTOR pathway is inhibited, and the maturation and antigen-presenting capacity of dendritic cells are weakened, indicating the pro-inflammatory role of HMGB1 in ALI
	Sivakorn et al. [57]	Prospective cohort study		Plasma concentrations of HMGB1 are significantly elevated in critically ill COVID-19 patients, especially in those who do not survive (*p* < 0.001), and are associated with worse SOFA scores (>10), septic shock, and adverse outcomes such as acute kidney injury	—	HMGB1 holds significant potential as a biomarker for predicting disease progression in critically ill COVID-19 patients
IL-1 RA	Gander-Bui et al. [61]	Animal model	Monocytes and macrophages	IL-1Ra exerts anti-inflammatory effects by blocking neutrophil recruitment and weakening pathogen containment; its treatment can restore neutrophil function, correct maladaptive hyperinflammatory responses, and clear other fatal infections	IL-1Ra	IL-1Ra secreted by macrophages can serve as a biomarker for infection
	Dahmer et al. [62]	Retrospective cohort study		IL-1Ra levels are significantly associated with the occurrence of pediatric ARDS (OR 1.30, 95% CI 1.10-1.52, *p* = 0.002) and independently predict mortality on day 1 (*p* = 0.02)	IL-1Ra	High levels of IL-1Ra are positively correlated with poor prognosis in ARDS
IL-10	Wang et al. [11]	Animal model	Macrophages	In ARDS patients, the ratio of Fth1 to Prok2 expression in pulmonary neutrophils is elevated, suggesting that the Fth1hiNeutrophi subpopulation may contribute to the pathological progression of the disease	—	Inhibiting Th1 cell differentiation may be a potential therapeutic strategy
IL-22	Taghavi et al. [69]	Animal model	Innate lymphoid cells	In the high-dose LPS injury group, IL-22 treatment significantly reduced IL-6 (110.6 vs 527.1 pg/mL, *p* = 0.04), TNF-α (5.87 vs 25.41 pg/mL, *p* = 0.04), and G-CSF (95.14 vs 659.6 pg/mL, *p* = 0.01) levels in BAL fluid. In the low-dose LPS group, there was a reduction in protein leakage (0.15 vs 0.25 µg/µL, *p* = 0.03)	—	IL-22 treatment significantly reduced the number of inflammatory cells and pro-inflammatory cytokine levels (such as IL-6, TNF-α, and G-CSF) in bronchoalveolar lavage (BAL) fluid, while also decreasing the pathological damage score of lung tissue. This suggests that IL-22 has therapeutic potential for ARDS
IL-13	Liu et al. [71]	Animal model	ST2+ regulatory T cells	Mice with IL-33 deficiency in the ALI model exhibited higher mortality and more severe lung pathology (*p* < 0.05); supplementation with recombinant IL-33 protein significantly reduced IL-6, G-CSF, and MCP-1 levels in BALF and restored immune balance (*p* < 0.01)	Local delivery of recombinant IL-33 protein	Mice lacking IL-33 exhibit higher levels of pro-inflammatory cytokines and more severe lung damage. Supplementation with IL-33 improves survival and reduces inflammatory responses by promoting Tregs to secrete IL-13

Given the limitations of single cytokine-targeted therapies in fully addressing the complex pathophysiology of ARDS, combination therapies have shown greater promise. Data from the RECOVERY trial indicate that tocilizumab combined with corticosteroids significantly reduced 28-day mortality in patients with severe COVID-19 (31% vs 35%, *p* = 0.0028); however, the efficacy of tocilizumab alone was limited, and the drug may increase mortality risk in patients not receiving corticosteroids [99]. This suggests that tocilizumab alone may not significantly improve clinical outcomes, and combination therapy could provide more benefits. Notably, the JAK inhibitor baricitinib has also demonstrated significant benefits when used in combination therapy in RCTs [111]. A meta-analysis further confirmed that, compared with tocilizumab monotherapy or control groups, the risk of mortality for patients with COVID-19 treated with combined corticosteroids and tocilizumab was 0.74 (95% CI: 0.36–1.50) and 0.48 (95% CI: 0.31–0.74), respectively, outperforming monotherapy [112]. This underscores the importance of multi-target combination therapies or individualized interventions. However, recent studies also suggest that the effectiveness and safety of combination therapies still require further validation in large-scale RCTs. For instance, a recent clinical trial showed that combining the JAK inhibitor ruxolitinib with statins did not improve outcomes in patients with COVID-19 [113].

While cytokine-based treatment strategies show potential, most therapies currently lack validation in large-scale, multicenter RCTs, limiting their widespread clinical application. Ongoing large-scale clinical trials, such as the study of tocilizumab and baricitinib combination therapy in patients with severe COVID-19 (NCT05082714), the ESKA trial evaluating anakinra for ARDS (NCT05914454), and the COMODULATE trial considering individualized treatment for heterogeneous patient populations (NCT06311448), will provide more robust evidence for the clinical application of cytokine-based therapies.

To address these challenges, future research should focus on several areas. First, studies should further elucidate the specific roles and mechanisms of cytokines in different ARDS subtypes and stages of the disease, particularly the spatiotemporal dynamics of the cytokine network, to provide a theoretical basis for precision-targeted therapy. Second, research should focus on developing new biomarker systems to identify high-responder patient populations and optimize treatment decisions and individualized approaches, such as RIPK1, fibronectin, and MMPs [114,115]. Additionally, conducting multicenter, large-sample, high-quality RCTs is essential to validate and optimize the efficacy and safety of novel anti-cytokine drugs. Furthermore, research should explore combination therapies related to cytokine regulation, such as those combined with mechanical ventilation or extracorporeal membrane oxygenation, to enhance overall efficacy. Lastly, emerging therapies, such as stem cell therapy and CAR-T cell therapy, which are gaining traction in ARDS treatment research due to their potential in immune regulation and personalized therapy, should be further investigated [116,117,118].

In conclusion, while ARDS treatment still faces numerous challenges, advances in understanding cytokine regulation mechanisms and developing individualized treatment strategies promise significant breakthroughs in improving ARDS outcomes in the future. Exploring multi-target combination therapies, enhancing biomarker-guided personalized interventions, and optimizing and applying emerging therapies, such as stem cell and CAR-T cell therapies, will be key directions for future development, offering new hope for ARDS patients.
